# Myomectomy by Robotically Assisted Laparoscopic Surgery: Results at Foch Hospital, Paris

**DOI:** 10.3389/fsurg.2015.00040

**Published:** 2015-08-20

**Authors:** Jennifer Asmar, Marc Even, Marie Carbonnel, Julie Goetgheluck, Aurelie Revaux, Jean Marc Ayoubi

**Affiliations:** ^1^Foch Hospital, Suresnes, France

**Keywords:** myomectomy, robotic laparoscopy, laparotomy, laparoscopy

## Abstract

We reported an observational, retrospective chart review of 36 women who underwent robotic myomectomy at the Department of Obstetrics and Gynecology, Foch Hospital. Short- and long-term results were analyzed. We compared our results with literature data. Potential advantages and limits of robotic surgery in myomectomy are discussed.

## Introduction

Uterine myomas are common benign tumors encountered in 30% of women of reproductive age and even more beyond 49 years of age (1). The common symptoms reported by women who have fibroids are menorrhagia, pelvic pressure or pain, and urinary symptoms. Also, fibroids that occupy the uterine cavity are responsible of infertility (2), and complications during pregnancy, such as miscarriages. Earlier diagnoses and a tendency for delaying childbearing have increased the need for uterine-sparing techniques in the surgical treatment of fibroids. The only surgical treatment that allows future pregnancies is the myomectomy, which can be done by laparotomy, laparoscopy, and recently robotic surgery.

Traditionally, myomectomy was performed by laparotomy. Recovery following surgery took 4–6 weeks. Laparoscopic surgery is usually performed as out-patient surgery under general anesthesia and has revolutionized gynecologic surgery. Studies have suggested that laparoscopic myomectomy leads to lower morbidity rates, better anesthetic results, less adhesions, and faster recovery than does laparotomic myomectomy (3, 4). Yet, laparoscopy has also its limits, as the quality of sutures still remains uncertain (5) and it is sometimes impossible to operate on large, poorly accessible, and multiple fibroids (6, 7). The technical difficulties encountered with laparoscopies are responsible of long learning curves (6). Although hard to evaluate, the rate of uterine rupture appears to be around 1% for both techniques (8, 9).

Robotically assisted laparoscopy constitutes a solution capable of overcoming certain limits of standard laparoscopy, as it enables easier suturing and can overcome laparoscopy’s limits (accessibility, size, number of fibroids). Its learning curve is significantly shorter, especially for complex procedures (10), but the data available are limited. This paper therefore aims at sharing our own experience using robotic surgery for myomectomy.

## Materials and Methods

### Surgical procedure

The Da Vinci SI High Definition four arm robotic system was used to perform all the surgeries of the study. After induction of general anesthesia, the patient was put in a dorsal lithotomy position. A uterine positioning system was used to mobilize the uterus with an intrauterine canula. Then, three to four standard, 0.7 cm incisions (depending on the technical difficulty of the surgery) were made and ports were inserted for the robot’s camera and instrument arms. The robot was then docked: the tower, which contains the robot’s four arms (three that can hold surgical instruments: monopolar scissors, bipolar surgical clamp, needle holders and a fourth that holds the system’s 3-D cameras) was positioned directly over the patient during surgery. These arms are controlled by a computer that exactly replicates the movements exerted by the operating surgeon on the joystick. The surgeon sitting behind the console operated the robot’s controls while looking into a stereoscopic monitor that provides a high definition 3-D view of the surgical site. The robot’s four arms were manipulated by maneuvering two master joysticks for a fingertip control precision of movement. The surgeon had also access to foot pedals providing additional options, such as the ability to switch between two different energy sources. Robotically assisted laparoscopic myomectomy was performed using the monopolar scissors and the bipolar grasping device, while sometimes using the fourth arm of the robot for myoma traction. After the incision in the uterus, the fibroid was excised and extracted from the surrounding uterine tissue. Finally, the uterus was sutured before removing the robot. The myomectomy site was sutured in one myometrial planes, usually by *x*-shape Vicryl 1^®^ stitches, and the uterine serosa was sutured by 4.0 monofilament overedge stitches in order to reduce the risk of adhesion. Then, a morcellator was placed and used to cut the fibroid into smaller pieces inside the patient’s abdomen after withdrawing the robot arms for more safety. Finally, these pieces were removed through one of the ports. An anti-adhesion gel was applied at the end of procedure in some cases.

### Study and patients description

The study was conducted as an observational, retrospective chart review. All the women diagnosed with uterine myomas who underwent robotic surgery from 1/2011 to 10/2014 were eligible for inclusion. Polymyomectomy for three myomas or more, myoma size exceeding 12 cm or suspicion of malignity were generally excluded because laparotomy was performed in these cases. But, we realized by robotic surgery cases to establishing the limits of robotic surgery. This explains that some cases had more than three myomas and size ranged up to 15 cm. In theses cases, patients were informed of a significant risk of laparoconversion. Oral and written informed consent about myomectomy and robotic surgery was given for each patients. Risks and controversies about morcellation were explained. Information concerning age, BMI, number of fibroids, localization, details of surgery, and direct complications was collected by screening patients’ files (surgery, anesthesia, and hospitalization reports). Information concerning long-term complications and consequences of surgery was collected by follow-up phone calls, at least 6 months after surgery. During the study period, 36 women underwent robotic myomectomy at the Department of Obstetrics and Gynecology, Foch Hospital.

Mean age was 39.1 years (21; 55). Six women were operated for treating infertility with fibroids distorting the uterine cavity, 24 because of pelvic pains associated with dysfunctional uterine bleeding (DUB), 5 for urinary disorders, and 1 because of pelvic distension. Average gestity was 1.13 (0; 8), average parity was 0.84 (0; 6). Ten of our patients had prior abdominal or pelvic surgery, including six laparotomies.

## Results

The average duration of the procedure was 161 min (65; 308). It was conducted under general anesthesia, as for laparoscopies, lasting 203 min (115; 375) on average. In 26 cases, 1 fibroid was removed, in 4 cases 2, in 3 cases 3, in 2 cases 4, and in 1 case 5 fibroids were removed. The mean weight and diameter of fibroids were 163.37 g (10; 400) and 73 mm (10; 150), respectively. In more than 70% of the cases (26/36), the uterus was sutured in two plans. Average blood loss during the surgery was 384 ml (50; 1000). No patients needed blood transfusion. For adhesion prevention, nine patients received Hyaloronic acid gel (Hyalobarrier^®^) and two of Icodextrine 4% solution (Adept^®^). Three cases of laparoconversion took place. One was necessary because of important bleeding during surgery after removing a 8 cm isthmic anterior myoma. In this case, laparotomic exploration showed that the hysterotomy was not completely sutured, and that the blood loss came from under the right broad ligament. Therefore, the surgeon achieved a ligature and section of the right round ligament and then the ligature and section of the right uterine artery after spotting the right ureter in order to achieve adequate hemostasis. The two others were because of the excessive size of the fibroids, exceeding 8 cm diameter (15 and 13 cm), and compelling the surgeon to use the laparotomic procedure to remove the myomas, because of the extreme technical difficulty of doing it by minimally invasive surgery with an increase blood loss. In one patient, a visceral wound (vesical) occurred during surgery, 1 cm long, sutured by three stitches.

### Short-term results

One immediate complication was reported at day 1 after surgery: a hemorrhagic duodenal ulcer that has required transfusion. The median hospitalization duration was of 3.29 days (1–12). Seven cases of anemia (with a hemoglobin rate of 10, 10.7, 8.6, 7, 8.8, 9.7, and 8.5) caused by excessive blood loss during surgery, were reported during hospitalization, none had required transfusion. One woman suffered of an abdominal scar sore, 15 days after her surgery, treated by only local treatment.

### Long-term results

After surgery, women were advised not to try to get pregnant before a delay of 6 months. Therefore, pregnancy data were only analyzed for women with a 6-month follow-up or more. Concerning women operated before April 2014 (32 women) and for which we had at least 6 months of follow-up, 5 patients desired a pregnancy, 4 obtained it. One got pregnant after 10 months, thanks to an ICSI procedure that had failed before the surgery. Another got pregnant after 6 months thanks to a FIV procedure (when six IIU had failed before the surgery), and two women got pregnant spontaneously, <6 months after the surgery. Unfortunately, one of these pregnancies ended up in a miscarriage, the second woman gave birth in October 2014.

No woman suffered from infection associated to the surgery, resistant pain, or had needed a rehospitalization for any reason. Only one woman had complaints about the esthetics of her scars.

## Discussion

Laparotomy is the traditional procedure used for myomectomy. It was first considered as a medical revolution as it enabled surgeons to remove fibroids avoiding hysterectomy and allowing women to consider future pregnancies. However, laparotomy was responsible of aftermath, such as adhesions (6) and heavy post surgery symptoms (blood loss, pain, long hospitalization duration.) (7).

Laparoscopy has clearly shown its short-term benefits in several studies, compared to laparotomy, such as reduced post operative pain, shorter hospital stay, and faster recovery. It also proved its similar therapeutic efficacy and safety (for myomas of ≤6 cm diameter and if their number did not exceed four myomas). Concerning long-term benefits, laparoscopy decreases adhesion rates (9).

However, technically, laparoscopy is a more difficult procedure for surgeons, especially when fibroids are not easily accessible or when >8 cm in size. Sutures are difficult to place and require well trained and experienced surgeons.

Concerning robotic surgery, procedure is easier than laparoscopy, thanks to easier sutures and access. The learning curve is significantly shorter in robotic surgery especially for complex procedures (10). Myomectomy through robotic surgery has been reported to be effective and safe since 2004 (11). In the retrospective study of Advincula et al. (12) that compared conventional laparotomy and robotic laparoscopy, the robotic surgery needed longer operating time: 231.38 ± 85.1 vs. 154.41 ± 43.14 min (*p* < 0.05) but with this technique blood loss and length of hospital stay were significantly reduced; laparotomy was associated with higher rates of morbidity.

The longer operating time associated with the robotic technique is related to the time necessary to set up the robot and to myoma extraction by morcellation.

The results of studies that evaluated robotic myomectomy vs. conventional laparoscopic myomectomy showed reduced blood loss and hospital stay, and conversion rates = 0 when laparoscopy was robotically assisted (12–16). In 2008, Ascher-Walsh et al. (17) have assessed 75 robotically assisted laparoscopic myomectomies vs. 50 conventional laparoscopic myomectomies: lengthened operating time was reported with the robotic technique (192 vs. 138 min) but blood loss was reduced (226 vs. 459 ml) and the duration of hospital stay was shorter (0.5 vs. 3.3 days). Even if short-term benefits of robotic assistance appear to be modest in standard myomectomy, in some complex cases and atypical locations (such as deep intramural fibroids, broad ligaments, uterine isthmus, anterior and posterior locations), the advantages of robotically assisted surgery allow overcoming laparoscopy limits (18, 19). In our study, the average blood loss was high: 384 ml. It is probably due to one case of important bleeding (1000 ml) with laparoconversion and some cases of big myomas (more than 10 cm). In these cases, robotic surgery is probably limited and laparotomy should be preferred.

Few studies reported on long-term side effects and benefit. Lonnersfors et al. describe a 68% pregnancy rate with a mean time to pregnancy (TTP) of 10 months. The study reported on 31 patients operated for an interstitial fibroid that deformed the uterine cavity. In 55% of cases, pregnancies were spontaneous (20). In a retrospective study on 872 women operated for myomectomy, Pitter et al. reported 107 pregnancies after surgery. This latter study reported that pregnancy chances and uterine rupture rates (1%) were similar in the laparoscopic vs. robotic surgery arms. Only 11% of women delivering by cesarean section had adhesions, which is less than the rates commonly encountered after surgery by laparoscopy. These numbers still need to be interpreted cautiously, as it only included few patients. The possible advantage of robotic surgery is primarily resting on the quality of sutures that might be similar to that of laparotomy, easily realizing two or three planes of adequate quality. In terms of long-term outcome, larger studies are needed to evaluate the rates of pregnancy after myomectomy by robotically assisted laparoscopy vs. laparotomy and the risk of secondary uterine rupture.

The cost of robotic myomectomy remains higher than those of conventional laparoscopy and laparotomy, with actual costs depending on reimbursement systems. In the US, Behera et al. (21) estimated the mean cost of robotically assisted myomectomy to be $7299, that of conventional laparoscopy $6219, and that of the abdominal route $4937.

Limits of robotic surgery still remain size and number of myomas. In our study, we reported two conversions for large myomas (13 and 15 cm). Myomas larger than 10 cm and more than 4 myomas should beneficiated a laparotomy procedure.

Another limitation of laparoscopy or robotic-assisted laparoscopy is the morcellation.

Minimally invasive gynecologic surgeons who perform laparoscopic intraperitoneal morcellation should be aware of the recent US Food and Drug Administration (FDA) warnings and possibility of litigation due to reports of intraperitoneal dissemination of cancerous cells. On November 24, 2014, the FDA issued a statement warning against using laparoscopic power morcellators in the majority of women undergoing hysterectomy or myomectomy for uterine fibroids (22). Morcellation should be performed within a contained environment to minimize any potential tumor spread in the event of an undiagnosed malignancy (23).

## Conclusion

Robotic myomectomy appears to reduce morbidity rates, offers better esthetic results, causes less adhesions, and features faster recovery as compared to myomectomy performed by regular laparoscopy. Compared with laparoscopy, robotic surgery allows to remove larger and less accessible myomas. The quality of sutures seems to equal that achieved by laparotomy, even if insufficient data on uterine rupture are currently available. Moreover, the possibilities of robotic surgery are bound to support the spreading of minimally invasive surgery. However, the price of robotic surgery still restrains its access. Ultimately, randomized clinical trials (RCTs) are urgently needed to support with data the recent tendencies observed in favor of robotic surgery.

## Conflict of Interest Statement

The authors declare that the research was conducted in the absence of any commercial or financial relationships that could be construed as a potential conflict of interest. The Review Editor, Jean-Bernard Dubuisson, declares that, despite having co-authored a manuscript with the author, Jean Marc Ayoubi, the review process was handled objectively.
